# Prognostic value of inflammatory cytokine levels, clinical efficacy, and prognosis of the hybrid artificial liver support system in chronic liver failure treatment

**DOI:** 10.5937/jomb0-55735

**Published:** 2025-10-28

**Authors:** Lihua Liu, Xiaoling Li, Juan Wang, Jing Ma, Xueping Nan

**Affiliations:** 1 Xi'an International Medical Center Hospital, Department of Gastroenterology, Xi'an, Shaanxi Province, China

**Keywords:** TNF-a, PCT, IFN-g, IL-2, IL-6, IL-10, chronic liver failure (CLF), plasma exchange (PE), double plasma molecular adsorption system (DPMAS), adverse reactions, survival rate, TNF-a, PCT, IFN-g, IL-2, IL-6, IL-10, hronična insuficijencija jetre (CLF), plazmafereza (PE), sistem za dvostruku adsorpciju molekula plazme (DPMAS), nepovoljne reakcije, stopa preživljavanja

## Abstract

**Background:**

This study aimed to evaluate the effectiveness and safety of the hybrid artificial liver support system (HALSS) in patients with chronic liver failure (CLF). It also sought to analyze the inflammatory response and patient prognosis.

**Methods:**

A total of 126 CLF patients were divided into three treatment groups: plasma exchange (PEG), double plasma molecular adsorption system (DPMASG), and a combination therapy group (CG). Key parameters, including liver and kidney function, blood coagulation, T lymphocyte subgroups, and inflammatory cytokine levels (TNF-a, procalcitonin [PCT], interferon-gamma [IFN-g], IL-2, IL-6, and IL-10), were assessed before and after treatment. The clinical efficacy, adverse reactions, and short-term prognosis were compared across the groups.

**Results:**

Compared to PEG and DPMASG, the combination group (CG) showed significant improvement in liver and kidney function markers, including reduced ALT, AST, total bilirubin (TBil), creatinine (Cr), INR, and prothrombin time (PT). Additionally, CG demonstrated increased levels of cholinesterase (ChE), albumin, and prothrombin activity (PTA). The CG group had a higher total clinical efficacy (92.0%) compared to PEG (76.3%) and DPMASG (78.9%). It also showed a lower rate of adverse reactions (8.0%) and improved one-year survival (36.0% vs. 18.4% and 21.1%, respectively). Furthermore, CG had the most favourable effects on inflammatory cytokine levels and T lymphocyte subsets, significantly reducing TNF-a, PCT, IFN-g, IL-2, IL-6, and IL-10.

**Conclusions:**

The combination of PEG and DPMAS (HALSS) demonstrated superior clinical efficacy in improving liver and kidney function, reducing inflammation, and enhancing patient prognosis compared to single therapies. These findings support HALSS as a promising adjunctive therapy for CLF patients, improving short-term outcomes and long-term survival.

## Abstract (sr)

**Uvod:**

Ova studija je imala za cilj da proceni efikasnost i bezbednost hibridnog sistema za podršku jetri (HALSS) kod pacijenata sa hroničnom insuficijencijom jetre (CLF). Takođe je nastojala da analizira inflamatorni odgovor i prognozu pacijenata.

**Metode:**

Ukupno 126 pacijenata sa CLF je podeljeno u tri grupe za lečenje: grupu sa plazmaferezom (PEG), grupu sa sistemom za dvostruku adsorpciju molekula plazme (DPMASG) i grupu sa kombinovanom terapijom (CG). Ključni parametri, uključujući funkciju jetre i bubrega, koagulaciju krvi, podgrupe T limfocita i nivoe inflamatornih citokina (TNF-a, prokalcitonin ŠPCTĆ, interferon-gama [IFN-g], IL-2, IL-6 i IL-10), procenjivani su pre i posle tret-mana. Klinička efikasnost, nepovoljne reakcije i kratko -ročna prognoza upoređene su među grupama.

**Rezultati:**

U poređenju sa PEG i DPMASG, kombinovana grupa (CG) pokazala je značajno poboljšanje markera funkcije jetre i bubrega, uključujući sniženje ALT, AST, ukupnog bilirubina (TBil), kreatinina (Cr), INR i protrom-binskog vremena (PT). Pored toga, KG je pokazala pove -ćane nivoe holinesteraze (ChE), albumina i aktivnosti pro-trombina (PTA). Ukupna klinička efikasnost u CG bila je viša (92,0%) u poređenju sa PEG (76,3%) i DPMASG (78,9%). Takođe je imala nižu stopu nepovoljnih reakcija (8,0%) i poboljšano jednogodišnje preživljavanje (36,0% naspram 18,4% i 21,1%). Dalje, CG je imala najpovoljnije efekte na nivoe inflamatornih citokina i podgrupe T limfocita, značajno smanjujući TNF-a, PCT, IFN-g, IL-2, IL-6 i IL-10.

**Zaključak:**

Kombinacija PEG i DPMAS (HALSS) pokazala je superiornu kliničku efikasnost u poboljšanju funkcije jetre i bubrega, smanjenju zapaljenja i unapređenju prognoze pacijenata u poređenju sa pojedinačnim terapijama. Ovi nalazi podržavaju HALSS kao dopunsku terapiju za paci-jente sa CLF koja obećava, poboljšavajući kratkoročne ishode i dugoročno preživljavanje.

## Introduction

Liver failure (LF) is a critical and often fatal clinical syndrome resulting from severe liver diseases, with a poor prognosis and high mortality rates. Chronic liver failure (CLF) is most commonly triggered by both viral and non-viral factors, with viral infections playing a major role in the progression of the disease. These infections lead to complications such as portal hypertension, spontaneous bacterial peritonitis, and, eventually, CLF [1] [2]. As CLF advances, it is marked by severe liver dysfunction, significant metabolic disturbances, and the activation of inflammatory cytokines, which further exacerbate liver injury and fibrosis [3]. While anti-infection therapies, liver protection strategies, nutritional support, and foundational treatments can slow disease progression, the prognosis remains poor in patients who are not eligible for liver transplantation, with mortality rates surpassing 80% in such cases [4] [5] [6].

The artificial liver support system (ALSS) has emerged as an effective therapeutic intervention for LF. This technology uses an extracorporeal device to temporarily replace liver function, providing crucial support to stabilize patients, buy time for recovery, or extend survival while awaiting liver transplantation [7]. Given the challenges of organ donation, the high costs associated with transplantation, and complications such as graft rejection, ALSS offers a promising alternative in clinical settings. However, it does notfully replace the need for liver transplants.

In recent years, advancements in ALSS technology have demonstrated significant benefits in treating CLF by shortening disease duration, reducing mortality rates, and improving patient outcomes. ALSS treatment modalities include plasma exchange (PE), dual-phase molecular adsorbent recirculating system (DPMAS), and hemodialysis filtration (HDF), with various combinations of these techniques yielding differing therapeutic results. Notably, the combined use of PE and DPMAS has shown superior outcomes compared to monotherapy, enhancing both liver and kidney function in CLF patients [8] [9]. This article reviews the clinical effects of PE, DPMAS, and PE+DPMAS-based ALSS therapies in managing CLF, exploring their impact on liver and kidney function and providing a basis for evaluating their safety, effectiveness, and potential long-term benefits in improving patient prognosis.

## Materials and methods

### Subjects

A total of 126 patients with chronic liver failure (CLF) from Xi’an International Medical Center Hospital were enrolled between January 2022 and January 2024, following the diagnostic criteria outlined in the *Guidelines for Diagnosis and Treatment of Liver Failure* (2018 edition). The patients were divided into three groups based on the treatment method: PEG (n=38), DPMASG (n=38), and the control group (CG, n=50).

Inclusion criteria:

1. Diagnosis of CLF according to the established criteria.
2. Age ≥ 18 years.
3. Indications for combined artificial liver therapy.
4. Complete clinical data available.
5. Expected survival of at least 6 months.

Exclusion criteria:

1. Diagnosis of primary or secondary liver cancer.
2. Liver failure induced by prior liver surgery.
3. Presence of immune deficiency.
4. History of liver or kidney transplantation or severe renal dysfunction.
5. Mental disorders prevent cooperation with the treatment protocol.
6. Pregnant or lactating women.

All patients provided informed consent, and the Xi’an International Medical Center Hospital Ethics Committee approved the study.

### Treatment

All patients accepted reduced glutathione (manufacturer: Shanghai Fudan Forward Pharmaceutical Co., Ltd., approval number: H20031265, 0.6 g) and polyene phosphatidyl choline (Chengdu Tiantai Mountain Pharmaceutical Co., Ltd., approval number: H20057684, 5 mL: 232.5 mg) injection. Before artificial liver treatment, a catheter with a single needle and dual lumen was placed in the femoral vein to establish femoral vein access, and the patient’s vital signs were continuously monitored during the treatment process. The patient was given 5 mg dexamethasone acetate (Shanghai General Pharmaceutical Co., Ltd., approval number: H31021298, 0.5 mL: 2.5 mg) and 1 g calcium gluconate (Sanchine, Harbin Pharmaceutical Group, approval number: H23021140, specification: 10 mL: 1 g) to prevent allergy before treatment.

PEG adopted plasma separator MICROPLAS MPS for plasma separation, displacement of plasma in 2,200–2,800 mL/min, the blood flow velocity of 90–120 mL/min, plasma separation speed 25 to 28 mL/min. The same amount of fresh frozen plasma was adopted as a supplement and was reinfused to the patient through the blood venous end circuit. This process was a comprehensive plasma exchange operation. PE was performed for about 1.5 hours, once a day, at intervals of 1–2 days, 2 to 3 times in total.

DPMASG adopted MICROPLAS MPS plasma separator. The plasma was connected to the BRS350exchange resin (cholesterol absorption column), and the HA330-II central porous resin (plasma irrigator), with a plasma treatment volume of 6 L.DMPAS treatment was carried out for approximately 2 hours, once a day, with intervals of 1 to 2 days, 2 to 3 times in total.

The plasma of patients in CG was separated by MICROPLAS MPS plasma separator, and PE was performed according to the method in PEG. After the replacement, according to the DPMAS set of methods for the adsorption process. The adsorbed plasma was transfused back to the patient through the venous circuit. PE combined with DPMAS was carried out for about 4 hours, once a day, at intervals of 1–2 days, 2–3 times in total.

### Evaluation of curative effect

Marked effectiveness: the patients’ clinical symptoms were visibly improved, the TBil was reduced by more than 50%, and the liver function was visibly improved. Effective: clinical symptoms improved, TBil was reduced by <50% and liver function improved also. Ineffective: no change or aggravation of clinical symptoms, no visible decrease or increase of TBil, no change or aggravation of liver function.

### Observation indicators

### (1) Peripheral blood-related indicators detection

5 mL of elbow venous blood was collected before treatment (0 mo) and 3 months after treatment (3 mo). The automatic biochemical analyzer was adopted for testing ALT, AST, ChE, Cr, total biliaryacid, albumin, INR, PT, and PTA. The CD4+ and CD8+ T cell subsets were detected by flow cytometry, and the CD4+/CD8+ ratio was calculated. An en - zyme-linked immunosorbent method was employed to detect inflammatory cytokine levels, and kits were bought from Shanghai Beyotime Biotechnology Co., LTD.

### (2) Prognosis assessment

At 0 mo and 3 mo, MELD (10) and CTP (11) were adopted to evaluate the prognosis of patients. The Cr, INR, and TBil values were entered into the MELD, and the MELD score was calculated according to the following equation.

MELD=3.8×ln[TBil(mg/dl)]+11.2×ln(INR)+9.6×ln[Cr(mg/dl)]+6.4×(0 or 1) (1)

0 means alcoholic primary disease, 1 means other types of primary disease. The anticipated mortality increases with a higher MELD score. CTP evaluation, according to hepatic encephalopathy for staging: stages 0, 1–2, and 3–4, ascites: no, moderate, and severe, TBil (μmol/L): <34.2, 34.2–51.3, and >51.3, PT (s): <4, 4–6, and >6, Alb (g/L): <28, 28–35, and >35, as1, 2, and 3 points, respectively, with 15 points as the maximum. The severity of the patient’s condition increases with a higher score.

### (3) Adverse reactions

The incidence of adverse reactions such as rash, pruritus, shivering, anaphylactic shock, hypotension, and hemolysis was recorded during the remedy.

### (4) Follow-up

A 12-month follow-up was carried out by telephone or outpatient clinic to record the overall SR of the patient.

### Statistical processing

SPSS 23.0 statistical software was employed. The constituent ratio described Count data, and the Chi-square test was adopted. The mean ± sd described measurement data and the t-test was adopted. Kaplan-Meier (KM) survival curve was drawn to compare the overall SR of subjects followed up for 12 months. *P*<0.05 was considered statistically meaningful.

## Results

### Contrast of general data in the subjects

Table 1 presents general data on CLF patients. Gender, age, primary disease, ascites, hepatorenalsyndrome, hepatic encephalopathy, and gastrointestinal bleeding had no visible distinction among the PEG, DPMASG, and CG (*P*>0.05).

**Table 1:** Contrast of general data in the subjects.

Information	PEG	DPMASG	CG
Gender [n(%)]			
Male	25 (65.8)	24 (63.2)	33 (66.0)
Female	13 (34.2)	14 (36.8)	17 (34.0)
Age (years)	48.9±8.2	49.1±9.9	48.8±10.1
Primary disease [n(%)]			
Alcoholic	8 (21.1)	9 (23.7)	13 (26.0)
Drug	4 (10.5)	4 (10.5)	6 (12.0)
Primary	1 (2.6)	1 (2.6)	1 (2.0)
Viral	25 (65.8)	24 (63.2)	30 (60.0)
Ascites [n(%)]			
Yes	33 (86.8)	35 (92.1)	43 (86.0)
No	5 (13.2)	3 (7.9)	7 (14.0)
Hepatorenal syndrome [n(%)]			
Yes	26 (68.4)	25 (65.8)	33 (66.0)
No	12 (31.6)	13 (34.2)	17 (34.0)
Hepatic encephalopathy [n(%)]			
Yes	28 (73.7)	27 (71.1)	35 (70.0)
No	10 (26.3)	11 (28.9)	15 (30.0)
Gastrointestinal bleeding [n(%)]			
Yes	3 (7.9)	4 (10.5)	5 (10.0)
No	35 (92.1)	34 (89.5)	45 (90.0)

### Contrast of effectiveness

**Figure 1:**
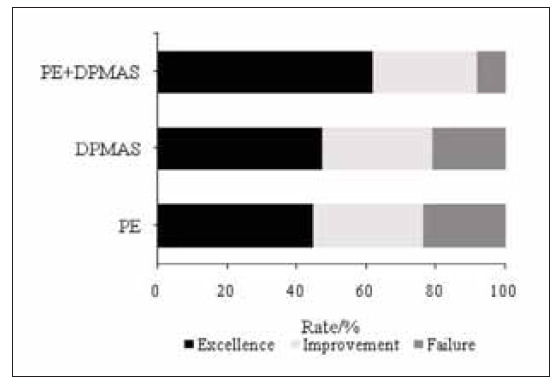
Clinical curative effect.

PEG: excellence in 17 subjects (44.7%), improvement in 12 subjects (31.6%), failure in 9 subjects (23.7%), total effective rate was 76.3% (29 subjects); DPMASG: excellence in 18 subjects (47.4%), improvement in 12 subjects (31.6%), failure in 8 subjects (21.1%), total effective rate was 78.9% (30 subjects); CG: excellence in 31 subjects (62.0%), improvement in15 subjects (30.0%), failure in 4 subjects (8.0%), total effective rate was 92.0% (46 subjects). The total effective rate was higher in CG relative to PEG and DPMASG (*P*<0.05) (Figure 1).

### Contrast of liver and kidney function in subjects

**Figure 2:**
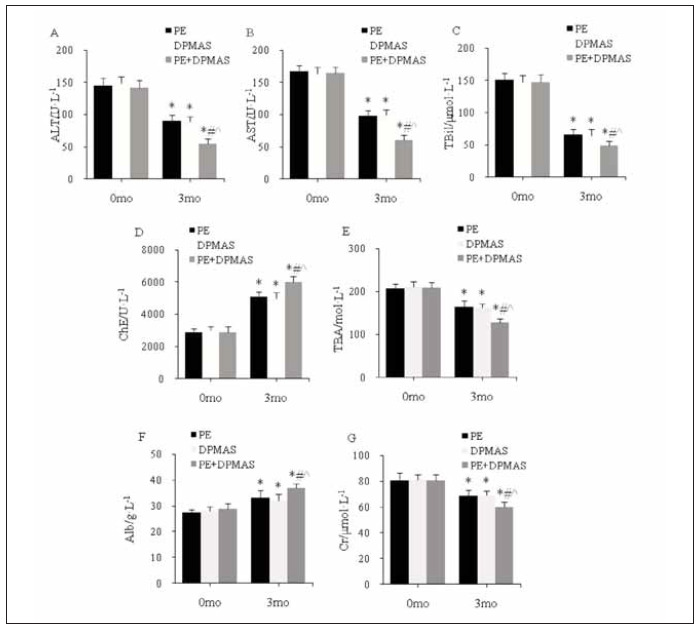
Peripheral blood and kidney function index level<br>(A–G: ALT, AST, TBil, ChE, TBA, Alb, Cr; *as against the same group at 0 mo, # as against PEG, ^ as against DPMASG, P<0.05)

As against the same group at 0 mo, at 3 mo, ALT, AST, TBil, TBA, Cr in peripheral blood were visiblyreduced, ChE and Alb were visibly increased in PEG, DPMASG, and CG; as against PEG and DPMAS Gat3 mo, ALT, AST, TBil, TBA, Cr in peripheral blood were visibly reduced, ChE and Alb were visibly increased in CG (all *P*<0.05) (Figure 2).

### Blood coagulation function in subjects

**Figure 3:**
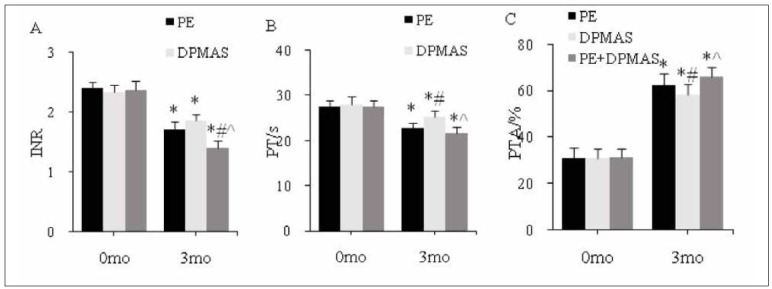
Peripheral blood clotting function index level.<br>(A–C: INR, PT, and PTA; *as against the same group at 0 mo, # as against PEG, ^ as against DPMASG, P<0.05)

As against the same group at 0 mo, at 3 mo, PT and INR in peripheral blood decreased visibly, and PTA was visibly increased in PEG, DPMASG, and CG; as against PEG, PT in peripheral blood was visibly increased, and PTA was visibly reduced in DPMASG at 3 mo; as against PEG and DPMASG, INR in peripheral blood was visibly reduced in CG at 3 mo; as against DPMASG, PT in peripheral blood was visibly reduced, and PTA was markedly increased in CG at 3 mo (all *P*<0.05) (Figure 3).

### T lymphocyte subsets

**Figure 4:**
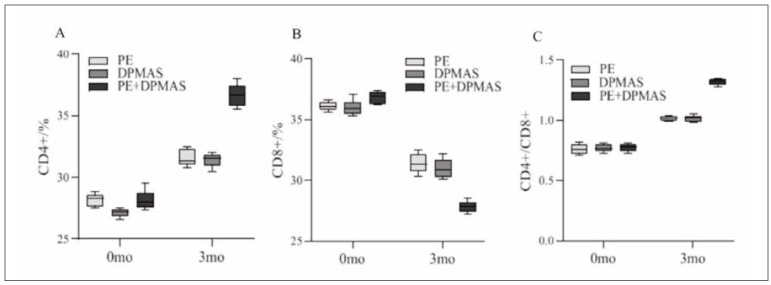
Peripheral blood T lymphocyte subgroup index level.<br>(A–C: CD4^+^, CD8^+^, and CD4^+^/CD8^+^)

As against the same group at 0 mo, CD4+ and CD4+/CD8+ in peripheral blood increased markedly, and CD8+ was decreased significantly in PEG, DPMASG, and CG at 3 mo; as against PEG and DPMASG, CD4+ and CD4+/CD8+increased markedly, and CD8+ was markedly decreased in CG at 3 mo (all* P*<0.05) (Figure 4).

### Inflammatory reaction in subjects

**Figure 5:**
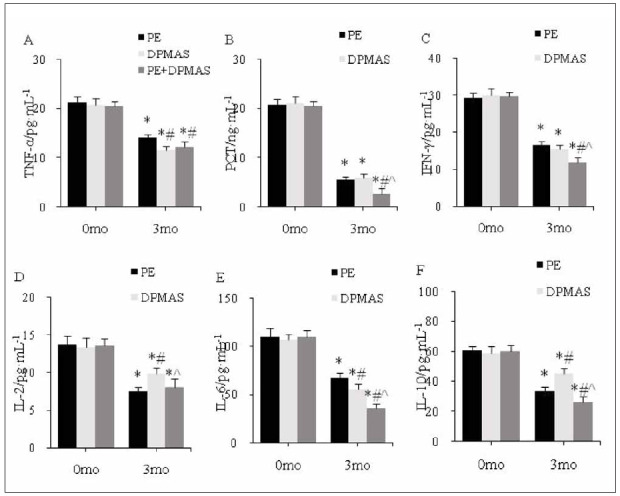
Peripheral inflammation index level.<br>(A–F: TNF-α, PCT, IFN-γ, IL-2, IL-6, IL-10; *as against the same group at 0 mo, # as against PEG, ^ as against DPMASG, P<0.05)

As against the same group at 0 mo, TNF-α, PCT, IFN-γ, IL-2, IL-6, and IL-10 in peripheral blood were markedly reduced in PEG, DPMASG, and CG at 3 mo; as against PEG, IL-2 and IL-10 increased markedly, and IL-6 was markedly reduced in DPMASG at 3 mo; as against PEG and DPMASG, TNF-α, PCT, IFN-γ, IL-6, and IL-10 were reduced significantly in CG at 3 mo (all P<0.05) (Figure 5).

### Prognosis of patients with CLF

**Figure 6:**
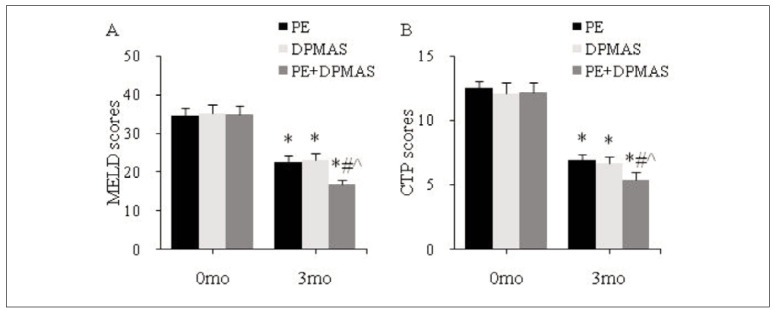
Prognostic indicators.<br>(A, B: MELD and CTP scores; *as against the same group at 0 mo, # as against PEG, ^ as against DPMASG, P<0.05)

As against the same group at 0 mo, MELD and CTP scores were markedly decreased in PEG, DPMASG, and CG at 3 mo; as against PEG and DPMASG, MELD and CTP scores were significantly reduced in CG at 3 mo (all *P*<0.05) (Figure 6).

**Figure 7:**
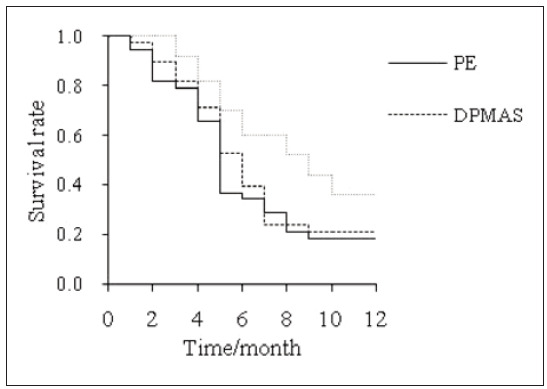
1-year KM survival curve.

In PEG, DPMASG, and CG, the1-year overall survival was 18.4%, 21.1%, and 36.0%, respectively. PEG and DPMASG had no visible distinction in the overall survival (*P*>0.05). The overall survival was markedly superior in CG relative to PEG and DPMASG (*P*<0.05) (Figure 7).

### Adverse reactions

The total probability of adverse reactions such as skin rash/pruritus, anaphylactic shock, chest tightness/palpitation, chills, hypotension, and hemolysis in PEG and DPMASG, and CG after treatment were 18.4%, 13.2%, and 8.0%, respectively. There was no clear difference in the total ARR between PEG and DPMASG (*P*>0.05). The total ARR of CG was clearly lower than that of PEG and DPMASG (*P*<0.05) (Table 2).

**Table 2:** Adverse reactions [n (%)].

Adverse reactions	PEG	DPMASG	CG
Skin rash/pru	3 (7.9)	2 (5.3)	2 (4.0)
Anaphylactic shock	1 (2.6)	0 (0.0)	1 (2.0)
Chest tightness/palpit	2 (5.3)	1 (2.6)	0 (0.0)
Chills	0 (0.0)	0 (0.0)	0 (0.0)
Hypotension	1 (2.6)	2 (5.3)	1 (2.0)
Hemolysis	0 (0.0)	0 (0.0)	0 (0.0)
**Total**	**7 (18.4)**	**5 (13.2)**	**4 (8.0)**

## Discussion

Patients with CLF have a wide range of pathological characteristics such as liver cell degeneration and necrosis, and fibrosis of liver tissue, which gradually progresses to critical severe LF [10] [11] [12]. Because of the large accumulation of endogenous toxins in the body of patients with CLF, it is highly likely to induce the development of complications such as abdominal fluid, liver disease, hepatic kidney syndrome, and gastrointestinal bleeding [13]. Degeneration and necrosis of large-range liver cells will affect normal liver function and cause metabolic disorders. Conventional medical treatment modes such as anti-infection, liver protection, and coagulation factor supplementation have not achieved good results in treating CLF. The treatment of liver transplants in CLF is excellent, but liver source deficiency, high transplant cost, and high post-transplant complications limit its clinical application [14].

An artificial liver support system is an essential auxiliary model currently used in CLF clinical remedy. It can effectively remove the patient’s body’s endogenous toxin and inflammatory mediators and provide a good internal environment for liver cell regeneration and recovery of normal liver function [15]. The appropriate mode (PE, DPMAS, bilirubin adsorption) can be chosen based on the actual situation. PE is common in CLF remedy mode. This technology can replace the plasma containing toxic substances in the patient’s body, then remove the accumulation of endotoxin, supplement bioactive substances such as immunoglobulin, reduce the generation of toxic metabolites, maintain the stability of the internal environment, and finally promote the recovery of liver function, improving the prognosis of patients [16] [17] [18]. DPMAS technology can specifically adsorb toxic substances such as bilirubin, bile acids, and inflammatory factors in patients’ plasma, reducing complications such as hepatic encephalopathy [19]. This article found that ALT, AST, TBIL, TBA, and Cr in the peripheral blood of patients after PE alone, DPMAS alone, and PE+DPMAS treatment were decreased, while ChE and Alb were increased, and the improvement of each index after PE+DPMAS treatment was the best. ALT, AST, TBA, and ChE are important indicators for assessing the function of the liver and can reflect liver injury and injury severity [20]. ALB is a protein that carries nutrients, regulates blood pressure, and maintains blood pH balance and other bodily functions. It is also an important indicator of liver synthesis function [21]. TBil> 170 mmol/L and PTA <40% are the important indices for diagnosis of LF [22]. Cr is a product of the metabolism of creatine. It is one of the indicators reflecting the state of renalfunction in patients [23]. PE, DPMAS, and PE+DPMAS modes can obviously improve the liver and kidney function of CLF patients, and the combination of PE+DPMAS has the optimal curative effect.

The disruption of T lymphocyte subsets plays a critical role in aggravating cellular immune dysfunction in liver failure (LF) patients, with this imbalance being closely associated with the severity of the disease [24]. In their study, Tang et al. [25] suggested that alterations in the number and metabolism of CD8+ T lymphocytes contribute to immune dysfunction in acute CLF, as observed in animal models. Likewise, Wang et al. [26] demonstrated that in HBV-infected acute CLF patients, peripheral blood T lymphocyte subsets were significantly altered, and these changes correlated with both the Prothrombin Time Activity (PTA) and the MELD score, proposing that T lymphocyte subset levels could serve as useful prognostic indicators for evaluating the progression of the disease. Consistent with these findings, our study observed that treatment with PE alone, DPMAS alone, and the combination of PE+DPMAS resulted in specific alterations in T lymphocyte subsets, with CD4+ T cells and the CD4+/CD8+ ratio increasing, while the CD8+ subset decreased. Notably, the combined PE+DPMAS treatment demonstrated the most substantial improvement in T lymphocyte subset balance, suggesting that this approach restored the immune cell population more effectively and enhanced the overall immune response in CLF patients. This improvement in immune function can be attributed to the combined regimen’s ability to promote the production and release of specific immune antibodies, which may help enhance the body’s immune defence mechanisms and potentially improve the prognosis of CLF patients. These findings align with previous studies, further supporting the therapeutic value of combined artificial liver support treatments in restoring immune homeostasis in liver failure.

Liver cell necrosis can trigger the production and release of many inflammatory factors, activating the body’s immune-inflammatory response and further migrating bone marrow granulocytes to the peripheral blood [27]. In addition, excessive inflammatory cytokines will stimulate the release of bone marrow monocytes and promote their migration to the liver. The infiltration of monocytes leads to local damage in liver tissue, induces the release of proinflammatory cytokines, injures the liver again, and forms a vicious cycle [28] [29]. IL-2 is a multipotent cytokine. The degree of hepatocyte injury is closely related to the level of IL-6 [30]. IL-10 can interact with cytokines and inhibit their production and release [31]. This article found that IL-6 in the peripheral blood of patients treated with DPMAS alone was lower than that of PE alone, while IL-2 and IL-10 were higher. Among them, PE+DPMAS treatment showed the best improvement in inflammatory cytokines. An artificial liver support system can improve the immune status of patients with LF by inhibiting Th1/Th2 balance.

MELD score can simply and objectively evaluate and predict the prognosis of patients with LF [32]. This article used MELD and CTP to evaluate patients’ LF prognosis. It was found that the MELD and CTP scores of patients treated with PE alone, DPMAS alone, and PE+DPMAS decreased. The MELD and CTP scores of patients treated with PE+DPMAS were lower, and the 1-year overall SR was higher. This is similar to the results of Yang et al. [33], who found that the 1-year SR of PE combined with DPMAS technology in CLF patients was as high as 45%. In addition, this article found that the ARR of patients treated with PE+DPMAS was markedly lower than that of patients treated with PE alone or DPMAS alone. PE can’t effectively remove inflammatory factors and Cr and other substances in the body of patients, so complications such as hypernatremia will occur after remedy, which cannot effectively improve the SR of patients [34]. PE+DPMAS remedy mode can improve the short-term prognosis of CLF, reduce the incidence of adverse reactions, and prolong the prospective SR.

One limitation of this study is its relatively small sample size, which may limit the generalizability of the findings to the broader population of CLF patients. Additionally, the study only assessed shortterm outcomes (3 months) and did not evaluate long-term effects or the potential for recurrence of liver failure. The study also lacked a detailed assessment of the mechanisms underlying the observed improvements, particularly regarding the inflammatory response and immune function. Lastly, the absence of a randomized controlled design introduces potential biases in treatment allocation, which may affect the reliability of the conclusions.

## Conclusion

In conclusion, the combination of PE and DPMAS as part of HALSS demonstrated superior clinicaloutcomes in patients with CLF compared to individual therapies. HALSS significantly improved liver and kidney function, reduced inflammatory cytokines, and enhanced immune responses, leading to better prognosis. The combination therapy resulted in a higher one-year survival rate and fewer adverse reactions, making it a promising option for CLF management. These findings support HALSS’s potential to improve both short-term outcomes and long-term survival in CLF patients.

## Dodatak

### Funding

The research is supported by Shaanxi Xi’an International Medical Center hospital research projects (No. 2021MS003).

### Conflict of interest statement

All the authors declare that they have no conflict of interest in this work.
